# Complement C3 Exacerbates Imiquimod-Induced Skin Inflammation and Psoriasiform Dermatitis

**DOI:** 10.1016/j.jid.2016.11.011

**Published:** 2017-03

**Authors:** Chiara Giacomassi, Norzawani Buang, Guang Sheng Ling, Greg Crawford, H. Terence Cook, Diane Scott, Francesco Dazzi, Jessica Strid, Marina Botto

**Affiliations:** 1 Centre for Complement and Inflammation Research, Department of Medicine, Imperial College London, London, UK; 2 Haemato-Oncology Department, King’s College London, UK

**Keywords:** IMQ, imiquimod, WT, wild type

To the Editor

The complement system is pivotal in protection against pathogens, but it also plays important roles in bridging innate and adaptive immune responses (Scott and Botto, 2016) and in modulating local and systemic inflammation (Markiewski and Lambris, 2007). Activation of complement occurs through three different pathways (classical, alternative, and lectin), converges at C3 cleavage, and culminates in the formation of the membrane attack complex. The anaphylatoxic fragments, C3a and C5a, generated during the proteolytic cascade, recruit immune cells that can promote the removal of debris and pathogens, but they can also cause tissue damage (Markiewski and Lambris, 2007).

The main source of complement is the liver. However, locally produced complement, particularly C3, can modulate inflammation in a variety of organs. There is also evidence that complement components are produced not only by immune cells such as macrophages and dendritic cells but also by nonimmune cells, which can contribute to local complement synthesis. In the skin, keratinocytes are a potential source of C3 (Pasch et al., 2000). A role for C3 in the skin has been identified in UV-induced immune tolerance (Hammerberg et al., 1998) and in the protection against T-cell–mediated inflammation (Purwar et al., 2011). Furthermore, locally produced C3 contributes to the inflammatory responses accompanying wound healing (Rafail et al., 2014), an effect mediated mainly through C5a/C5aR interactions. The latter also play an important role in influencing the skin microbiome (Chehoud et al., 2013). In keeping with these observations in animal models, complement has been shown to have a role in the pathogenesis of many human skin diseases, including psoriasis (Kotnik, 2011).

Psoriasis is an inflammatory skin disease characterized by epidermal hyperplasia, infiltration of immune cells, and secretion of inflammatory cytokines (Tortola et al., 2012). The anaphylatoxic fragments, C3a and C5a, have been found in corneal scale extracts from psoriatic lesions (Takematsu et al., 1986). The C5a from these lesions has been shown to chemoattract monocyte-derived dendritic cells (Mrowietz et al., 2001), indicating that complement may contribute to the inflammatory process in this disease. In the inducible AP-1–dependent psoriasis-like mouse model, the S100A8-S100A9 complex that promotes skin inflammation has been shown to up-regulate C3 expression (Schonthaler et al., 2013). Psoriasis-like dermatitis can be induced by topical application of the toll-like receptor 7 agonist imiquimod (IMQ) (van der Fits et al., 2009) in the form of Aldara cream (3M Pharmaceuticals, St Paul, MN) (Walter et al., 2013). Here, we explore the role of complement in IMQ-mediated psoriasiform dermatitis.

We first tested whether cutaneous IMQ treatment induces local C3 synthesis (experimental methods are provided as Supplementary Materials online) and found a progressive increase in C3 mRNA in the skin with repeated IMQ applications (Figure 1a, and see Supplementary Figure S1a online). Immunohistochemistry showed that the C3 expression was predominantly in the dermis (see Supplementary Figure S1b). In vitro experiments with isolated dermal stromal cells showed that these cells can produce C3 upon stimulation with inflammatory cytokines known to be induced in IMQ-treated skin but not upon direct challenge with a toll-like receptor 7 agonist (see Supplementary Figure S1c). To test whether C3 contributes to the psoriatic-like lesions induced by IMQ, we then treated wild-type (WT) and *C3*^*–/–*^ mice for 7 consecutive days. The treatment resulted in skin thickening, scaling, and erythema (Figure 1b). However, mice lacking C3 displayed less skin inflammation compared with WT mice (Figure 1c). Consistent with the reduced skin response in *C3*^*–/–*^ mice, we found significantly fewer infiltrating neutrophils, but slightly more monocytes, and no difference in the number of resident γδ T cells compared with WT animals (Figure 1d). IL-17 secretion by γδ T cells plays a key role in the IMQ-induced psoriasis model (van der Fits et al., 2009), and our data confirmed that IL-17 secretion was mainly restricted to these cells. The frequency of IL-17–positive γδ T cells in both skin and draining lymph nodes was significantly lower in the absence of C3 (Figure 1e and f). All animals were handled in accordance with institutional guidelines, and the UK Home Office approved the procedures.

We next evaluated whether C3 contributes to the local inflammatory response. To this end, we analyzed the gene expression of a selected number of cytokines/chemokines known to be induced in the skin by IMQ treatment (Di Meglio et al., 2014, Walter et al., 2013). At the peak of clinical inflammation on day 7, the *C3*^*–/–*^ mice did not show any obvious differences in inflammatory gene expression (Figure 2a). However, at the onset of the clinical pathology, day 3, when the cytokine/chemokine gene response peaks (Di Meglio et al., 2014), C3-deficient mice had a markedly reduced response, suggesting that C3 modulates the inflammatory gene induction that precedes clinical manifestations. To substantiate this, we carried out a time-course analysis of skin gene expression after 3 days of IMQ treatment. This showed that in the absence of C3, the resolution of the IMQ-triggered inflammation was faster compared with WT mice. Twenty-four hours after the last application, C3-deficient mice had significantly reduced levels of all genes analyzed, namely those for IL-1α, TNF-α, IL-17a, IL-23a, CXCL1, and CCL2 (see Supplementary Figure S2a and Supplementary Table S1 online). Consistent with this, thickening of the skin assessed by histology was significantly reduced in the *C3*^*–/–*^ mice (Figure 2b and c). The altered skin pathology in the IMQ-treated C3-deficient mice appeared to affect mainly the epidermis. Untreated skin of WT and *C3*^*–/–*^ mice was histologically indistinguishable.

In summary, we demonstrate that C3 is involved in the development and resolution of the psoriasiform skin inflammation induced by short-term treatment with IMQ. The proinflammatory effect of C3 is likely to be mediated by several mechanisms. In the absence of C3 the expression of psoriasis-relevant genes in the skin was impaired, neutrophil infiltration into the inflamed site was decreased, and IL-17 production by γδ T cells in the skin and the draining lymph nodes was reduced. Taken together, these data support a proinflammatory role of C3 during psoriasis-like skin inflammation.

## Conflict of Interest

The authors state no conflict of interest.

## Figures and Tables

**Figure 1:**
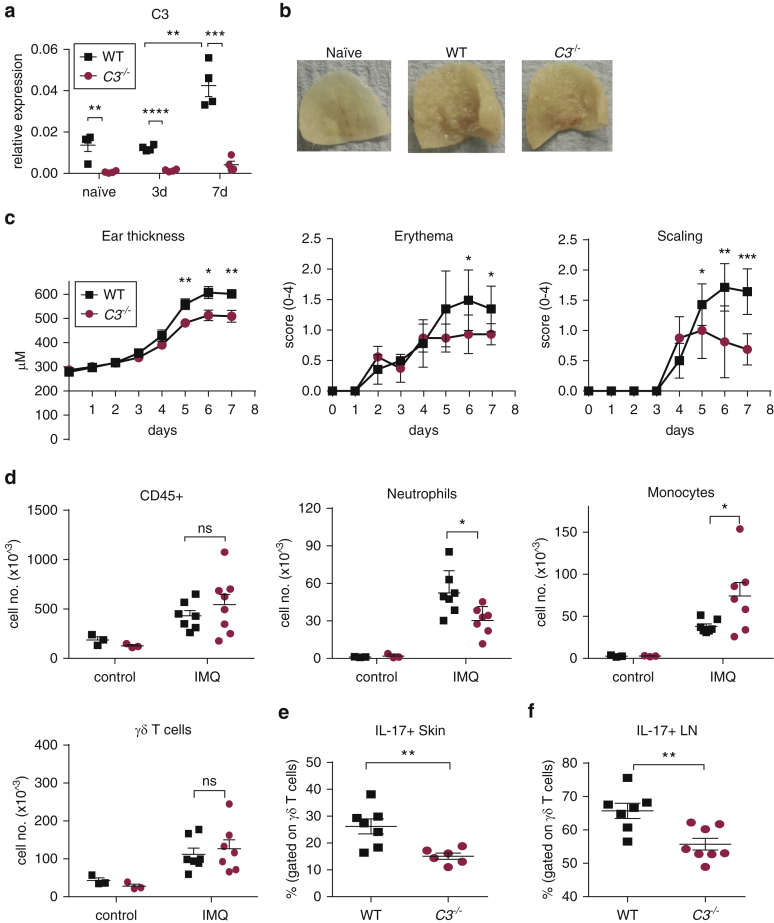
**IMQ-induced skin inflammation and psoriasiform dermatitis is impaired in C3-deficient mice.** IMQ was applied for 7 days to the ear of BALB/c WT (black square) and BALB/c.*C3*^–/–^ (red circle) mice. (**a**) C3 expression in whole skin quantified by quantitative real-time –PCR 24 hours after the last application (n = 4). Naïve indicates untreated mice, mean ± standard error of the mean relative to cyclophilin, unpaired *t* test. (**b**) Representative pictures of IMQ-treated ears at day 7. (**c**) Ear thickness (mean ± standard error of the mean) and clinical scores (mean ± standard deviation) for erythema and scaling. Representative experiment of two, Mann-Whitney test. (**d**) Number of CD45^+^ cells, neutrophils (Ly-6G^+^), monocytes (CD11b^+^Ly-6C^+^Ly-6G^–^), and γδ T cells (CD4^–^CD3^+^γδTCR^+^) at day 7. (**e–f**) Proportion of IL-17^+^ γδ T cells from (**e**) skin and (**f**) LN after in vitro restimulation. Mean ± standard error of the mean, unpaired *t* test. ^∗^*P* < 0.05, ^∗∗^*P* < 0.01, ^∗∗∗^*P* < 0.001, ^∗∗∗∗^*P* < 0.0001. d, day; IMQ, imiquimod; LN, lymph node; M, meter; no., number; WT, wild type.

**Figure 2:**
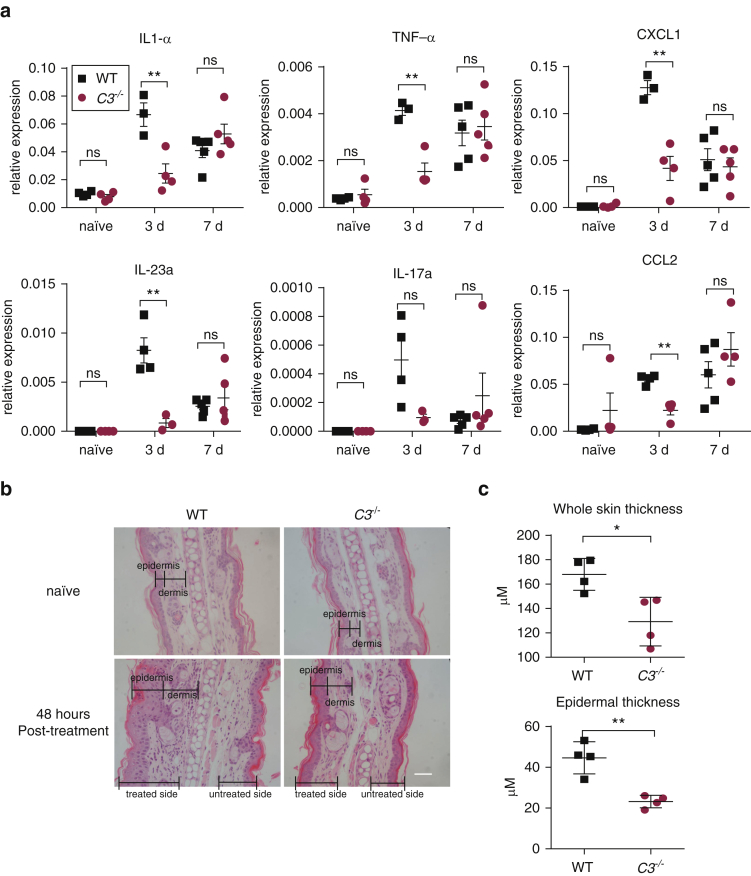
**Reduced early proinflammatory response and skin thickening in IMQ-treated C3-deficient mice.** (**a**) IMQ was applied for 3 or 7 days to the ear of BALB/c WT (black square) and BALB/c.*C3*^–/–^ (red circle) mice. Gene expression was analyzed in whole skin 24 hours after the last IMQ exposure (n = 3–5/group). Naïve indicates untreated mice, mean ± standard error of the mean relative to the control gene cyclophilin (SYBR Green assay, Life Technologies, Waltham, MA) or GADPH (Taqman assay ThermoFisher, Waltham, MA) levels. (**b**) Representative images showing hematoxylin and eosin staining of cross-sectional ear skin from BALB/c and BALB/c*.C3*^–/–^ mice untreated and 48 hours after the third IMQ application. The epidermis/dermis boundaries are indicated. Scale bars = 50 μm. (**c**) Quantification of whole-skin and epidermal thickness calculated using Image J software (National Institutes of Health, Bethesda, MD). Each symbol represents an individual mouse (n = 4). Mean ± standard error of the mean; unpaired *t* test. ^∗^*P* < 0.05, ^∗∗^*P* < 0.01. d, day; GAPDH, glyceraldehyde-3-phosphate dehydrogenase; IMQ, imiquimod; M, meter; ns, not significant; TNF, tumor necrosis factor; WT, wild type.
