# Supplementary material for: Complement C3 Exacerbates Imiquimod-Induced Skin Inflammation and Psoriasiform Dermatitis
Source: J Invest Dermatol. 2017 Mar;137(3):760–3. doi: 10.1016/j.jid.2016.11.011 (PMC5319416; doi:10.1016/j.jid.2016.11.011)
Supplement: Supplementary Data [file mmc1.pdf]

Supplementary information online

Title: Complement C3 exacerbates imiquimod-induced skin inflammation and psoriasiform dermatitis

Authors: [1]Chiara Giacomassi, [1] Norzawani Buang, [1]Guang Sheng Ling, [1]Greg Crawford, [1]H. Terence Cook, [1]Diane Scott, [2]Francesco Dazzi, [1,3]Jessica Strid, [1,3]Marina Botto

Institute: [1]Centre for Complement and Inflammation Research, Department of Medicine, Imperial College London, London, United Kingdom
[2] Haemato-Oncology Department, King's College London, United Kingdom
[3] Co-senior author

Correspondence address:

Professor Marina Botto
Centre for Complement and Inflammation Research,
Division of Immunology and Inflammation,
Department of Medicine,
Imperial College London, Hammersmith Campus,
Du Cane Road, London W12 0NN, UK
Tel.: +44-(0)20 3313 2316
Fax: +44-(0)20 3313 2379
E-mail: m.botto@imperial.ac.uk

Materials and Methods

Mice

Mice deficient in complement component C3 (*C3*$^{-/-}$) (Wessels et al., 1995) or C1q (*C1qa*$^{-/-}$) (Botto et al. 1998) were backcrossed onto the BALB/c background for more than 10 generations. BALB/c or C57BL/6 wild type (WT) mice were purchased from Charles River (UK). Experimental mice were 8-12 weeks of age, strain-, sex- and age-matched. All animals were handled in accordance with the institutional guidelines and the UK Home Office approved the procedures. The experiments with animals were conducted following the ARRIVE guidelines.

IMQ treatment

The *Aldara*® cream (3M Pharmaceutical - 5% Imiquimod) was applied for 7 consecutive days on the ventral side of the right ear. Skin thickening was assessed daily by measuring ear thickness using a micrometer (Mitutoyo, Japan). Erythema and scaling were measured blindly using a score ranging from 0 to 4 (0 = absence of clinical signs, 4 = severe phenotype). Mice were sacrificed after 7 topical applications. Treated ears were collected, digested as described below and cells stained for surface markers. Contralateral untreated ears were also collected and used as control.

Skin digestion

The ventral side of the ears were split mechanically before being minced and transferred to digestion buffer consisting of PBS, 0.13 Wünsch units/mL of Liberase TM Research grade (Roche), 25μl/ml of DNase I (Roche) for 90 minutes at 37°C with gentle shaking. After the enzymatic digestion, skin fragments were processed using a gentle MACS homogeniser (Milteny) and the cell suspension was filtered through a 70 μM cell strainer (BD). Cells were washed, resuspended in flow cytometry buffer (PBS/1%BSA) before staining.

Flow Cytometry

Cells were stained using standard protocols in the presence of saturating anti-FcγRII/III (2.4G2). The following antibodies were used: CD45 (30-F11), Ly-6c (HK1.4), CD11b (M1/70), all from e-Bioscience; TCRγδ (GL3), Ly-6G (1A8) CD45 (30-F11), CD90.2 (53-2.1) all from Biolegend; CD3e (500A2), CD4 (RM4-5), IL-17A (TC11-18H10), IFNγ (XMG1.2), Ly-6A/E (E13-161.7) CD45.2 (104) all from BD Pharmingen. Flow cytometry was performed with a BD FACSVerse (BD 40 Biosciences, CA, USA). Data were analysed using FlowJo software, version 7.6.5 (TreeStar Inc, Ashland, OR, USA).

In vitro stimulation and intracellular staining

Single cell suspensions from draining (dLN) or treated skin harvested at day 7 were plated in a U-bottom 96 well plate at 4x10^{5}cells/well in a final volume of 200 μl of RPMI 1640 (Gibco), 10% FBS, 100 U/mL penicillin, and 100 μg/mL streptomycin. Cells were stimulated *in vitro* with PMA (25ng/mL) (Sigma) and Ionomycin (Sigma)

(1 μg/ml) in the presence of Golgi stop (monensin-BD). After 4 hours cells were harvested, permeabilised/fixed with Cytofix/CytoPerm Kit (BD), according to manufacturer's instructions, and stained for intracellular IL-17A.

Gene expression analysis and histology after IMQ treatment

Mice were treated with 5 mg of Aldara® cream on the ventral side of the right ear for 3 or 7 consecutive days and the skin was analysed at different time points after the last treatment. Ear skin tissue was: i) processed for histology and stained with haematoxylin and eosin (H&E); ii) embedded in OCT (Tissue-Tek) and frozen immediately on dry ice; iii) stored in RNA later (Sigma Aldrich) at -20°C. C3 immunofluorescence: 8 μM frozen sections were fixed in acetone, blocked with 10% normal goat serum and stained with a FITC-labelled goat anti-mouse C3 antibody (Cappell/ICN, dilution of 1:100). Samples were analysed using a Leica SP5 confocal microscope. Gene expression analysis: RNA was extracted from whole skin using RNeasy mini kit (Qiagen), after homogenisation with TissueLyserII (Qiagen). RNA was quantified by absorbance spectroscopy (Nanodrop) and cDNA synthesized by reverse-transcriptase reaction (Bio-Rad). qRNA analysis was performed using the Real-time PCR Detection System and Power SYBR Green (ThermoFisher) or TaqMan system (ThermoFisher) (supplementary table S1). The data are expressed as relative expression to the Cyclophillin or the GAPDH housekeeping gene.

Isolation of dermal stromal cells and C3 secretion

Primary dermal stromal cells were isolated from C57BL/6 mice. 1x1 cm of shaved skin was excised, minced and digested in 10 ml of digestion buffer: DMEM/F12 media (Gibco), 0.13 Wünsch units/mL of Liberase TM Research grade (Roche) and 1x antibiotic/antimycotic (Life Technologies). After 2 hour incubation at 37°C, the skin fragments were washed, resuspended in 15% FBS DMEM/F12 medium and the cells transferred to culture flasks. The medium was changed every 3-4 days and the tissue fragments were removed after 1 week. When cells reached 70% to 80% confluence, the adherent cells were expanded into larger flasks. The cells were phenotyped by assessing the expression of CD11b, CD45.1, CD71, Sca-1 (Ly-6A/E), CD90.2 and PDGFR-α (CD140a) by flow cytometry. Cells were stimulated with IL-1β (100 ng/ml) or TNF-α (100 ng/ml) or IL-36 (100 ng/ml) or R848 (doses ranging from 0.1 to 1 mg/ml) for 24hrs. C3 levels were measured using as previously described (Ruseva et al., 2013).

Statistical Analysis

Comparison between two groups was performed using an unpaired two-tailed Student's *t* test. Non-parametric data were assessed using Mann-Whitney test. Results were expressed as mean ± SEM. *P* values less than 0.05 were considered statistically significant. Statistical analysis was performed using GraphPad Prism version 6.0 (GraphPad Software, San Diego, CA).

Table S1

Gene	Forward primer (5’ to 3’)	Reverse primer (5’ to 3’)
C3a	CAAACAGATGTCCCTGACCA	TGCTTTTGAGTTTGGCATGA
S100a9	GGAGCGCAGCATAACCACCATC	GCCATCAGCATCATACACTCCTCA
TNF-α	AGGGATGAGAAGTTCCCAAATG	CACTTGGTGGTTTGCTACGAC
IL1-α	TTGGTTAAATGACCTGCAACA	GAGCGCTCACGAACAGTTG
CXCL1	ACTCAAGAATGGTCGCGAG	GTGCCATCAGAGCAGTCTG
CCL2	CCCAATGAGTAGGCTGGAGA	CCTTAGGGCAGATGCAGTTT
Cyclophilin	CAAATGCTGGACCAAACACAA	CCATCCAGCCATTCAGTCTTG
**Gene**	**Taqman primer**	
IL-17a	Mm00439618_m1	
IL-23	Mm00518984_m1	
GADPH	Mm.304088	

References

Botto M, Dell’Agnola C, Bygrave AE, Thompson EM, Cook HT, Petry F, et al. Homozygous C1q deficiency causes glomerulonephritis associated with multiple apoptotic bodies. Nat Genet. 1998;19(1):56–9.

Ruseva MM, Vernon K a, Lesher AM, Schwaeble WJ, Ali YM, Botto M, et al. Loss of properdin exacerbates C3 glomerulopathy resulting from factor H deficiency. J Am Soc Nephrol. 2013 Jan;24(1):43–52.

Wessels MR, Butko P, Ma M, Warren HB, Lage a L, Carroll MC. Studies of group B streptococcal infection in mice deficient in complement component C3 or C4 demonstrate an essential role for complement in both innate and acquired immunity. Proc Natl Acad Sci U S A. 1995;92(25):11490–4.

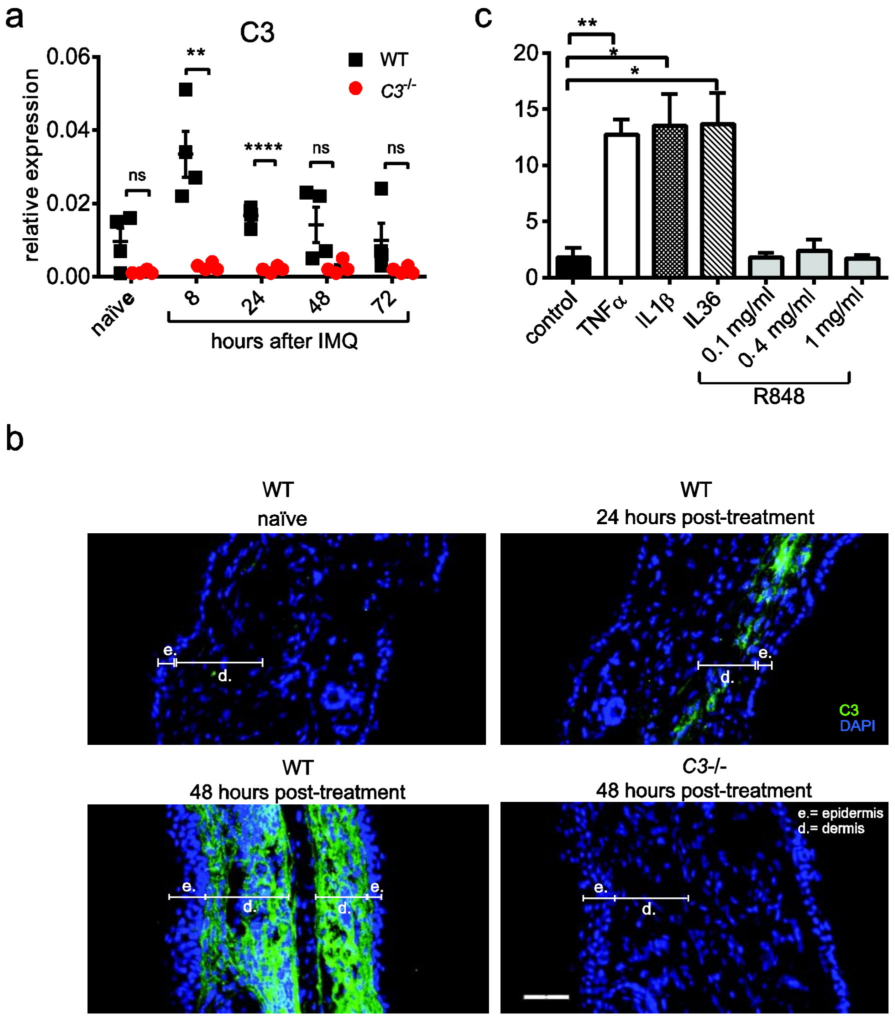

Figure S1. Cutaneous IMQ exposure promotes local C3 production.

IMQ was applied topically for 3 consecutive days to the ventral ear of BALB/c WT and BALB/c.*C3*$^{-/-}$ mice and (a) expression of C3 in whole ear skin was quantified by qRT-PCR at the indicated times after the last application (n=4). Untreated mice were used as controls. Data are expressed as mean ± SEM relative to cyclophilin. Results shown are representative of two independent experiments (*n*=3-4/group). p values by unpaired *t* test; ns=non-significant; **p <0.01; **** p <0.0001. (b) Representative images showing cross-sections of naïve and IMQ-treated skin stained with C3 (green) 24 and 48 hrs after the last IMQ treatment. Nuclei (blue) identified with DAPI. Scale bar represents 50 μM. (c) C3 secretion by dermal stromal cells following *in vitro* stimulation with the cytokines indicated or R848 (ranging from 0.1 to 1 mg/ml) for 24 hours. C3 measured by ELISA. Data are expressed as mean ± SEM, *p <0.05, **p <0.01 (unpaired *t* test).

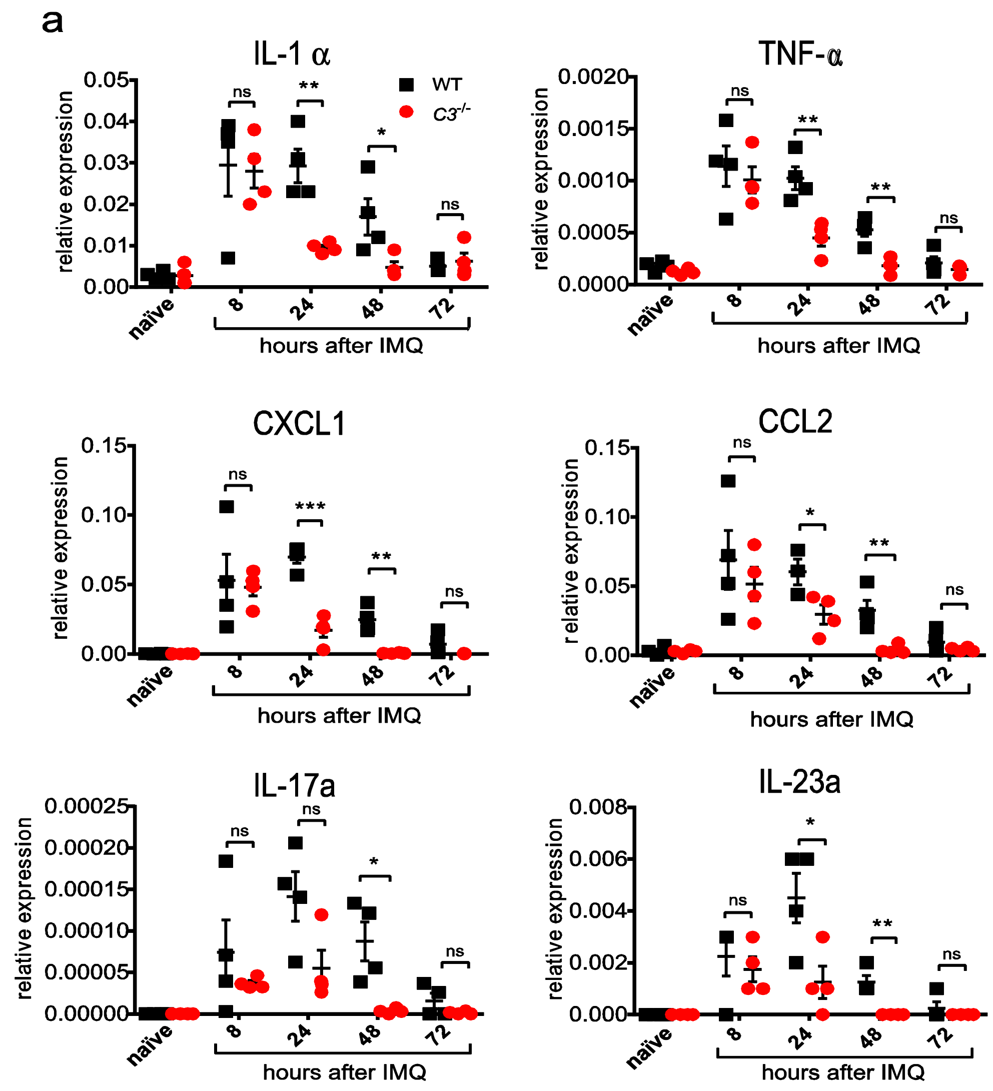

Figure S2. C3 deficiency accelerates the resolution of the inflammation triggered by IMQ.

IMQ was applied for 3 days to BALB/c WT and BALB/c.*C3*$^{-/-}$ mice. (a) Expression of psoriasis-relevant genes quantified by qRT-PCR at the indicated times after the 3rd application (n=4). Gene expression is relative to cyclophilin (SYBR Green assay) or GADPH (Taqman assay) levels. Each symbol represents an individual mouse (n=4). Data shown as mean ± SEM; unpaired *t* test; ns=non-significant; *p <0.05, **p <0.01; ***p <0.001. Naïve=untreated mice. Results representative of two independent experiments.
